# Platelets and Infections – Complex Interactions with Bacteria

**DOI:** 10.3389/fimmu.2015.00082

**Published:** 2015-02-26

**Authors:** Hind Hamzeh-Cognasse, Pauline Damien, Adrien Chabert, Bruno Pozzetto, Fabrice Cognasse, Olivier Garraud

**Affiliations:** ^1^GIMAP-EA3064, Université de Lyon, Saint-Etienne, France; ^2^Etablissement Français du Sang Auvergne-Loire, Saint-Etienne, France; ^3^Institut National de la Transfusion Sanguine, Paris, France

**Keywords:** platelet, inflammation, adhesion, infection, cytokine, chemokine, sepsis

## Abstract

Platelets can be considered sentinels of vascular system due to their high number in the circulation and to the range of functional immunoreceptors they express. Platelets express a wide range of potential bacterial receptors, including complement receptors, FcγRII, Toll-like receptors but also integrins conventionally described in the hemostatic response, such as GPIIb–IIIa or GPIb. Bacteria bind these receptors either directly, or indirectly via fibrinogen, fibronectin, the first complement C1q, the von Willebrand Factor, etc. The fate of platelet-bound bacteria is questioned. Several studies reported the ability of activated platelets to internalize bacteria such as *Staphylococcus aureus* or *Porphyromonas gingivalis*, though there is no clue on what happens thereafter. Are they sheltered from the immune system in the cytoplasm of platelets or are they lysed? Indeed, while the presence of phagolysosome has not been demonstrated in platelets, they contain antimicrobial peptides that were shown to be efficient on *S. aureus*. Besides, the fact that bacteria can bind to platelets via receptors involved in hemostasis suggests that they may induce aggregation; this has indeed been described for *Streptococcus sanguinis*, *S. epidermidis*, or *C. pneumoniae*. On the other hand, platelets are able to display an inflammatory response to an infectious triggering. We, and others, have shown that platelet release soluble immunomodulatory factors upon stimulation by bacterial components. Moreover, interactions between bacteria and platelets are not limited to only these two partners. Indeed, platelets are also essential for the formation of neutrophil extracellular traps by neutrophils, resulting in bacterial clearance by trapping bacteria and concentrating antibacterial factors but in enhancing thrombosis. In conclusion, the platelet–bacteria interplay is a complex game; its fine analysis is complicated by the fact that the inflammatory component adds to the aggregation response.

## Introduction

The molecular make-up of platelets, which are specialized in repair and in innate immunity (1–4), makes these “cells” unique blood elements. The recognition of platelets as cells is still controversial, primarily due to their lack of a nucleus. However, the multitude of functions that have recently been attributed to them and that are presented in this manuscript support our decision to consider them as such throughout these studies. The platelet response, which was once believed to only be involved in hemostasis, is in fact extremely complex and probably adapts when required. In this review, we will address the inflammatory potential of platelets when confronted with pathogenic invasion, and more specifically when it involves bacteria or viruses. We will focus on their ability to directly trigger an immune response, ranging from recognition of the pathogen to the orchestration of its elimination.

## Platelets and Bacterial Infections

### Platelets at the interface between bacterial infection and thrombosis

#### Example of infectious endocarditis

Cardiovascular diseases, although varied, may have infectious origins, as was described by Beynon et al. concerning infectious endocarditis (IE) (5). The main bacterial agents involved in IE are *Staphylococcus aureus*, *Streptococcus sanguinis*, and *Streptococcus gordonii (S. gordonii)*. According to epidemiological studies, bacteremia that leads to the development of this disease may be the consequence of a local intervention, but there may be a more distant origin such as the recurrent administration of a drug or a surgical dental procedure.

By creating an inflammatory environment, bacteria adhere to the valvular endothelium and increase its permeability, thus leading to the exposure of subendothelial tissue factors. The circulating platelets then adhere to the subendothelium, and their hemostatic activation causes the formation of a thrombus, which can then lead to arterial ischemia, and even pulmonary embolism (6). IE is therefore a disease that links inflammation and hemostasis, though it was long accepted that platelet activation occurred indirectly.

In IE, analysis of a newly formed thrombus in the myocardium showed the presence of bacteria inside the platelet clot (7). The first hypotheses suggested that this bacterial presence stabilized the platelet clot due to the activity of bacterial enzymes specialized in coagulation [coagulase for *S. aureus* (8) or the “clumping factor” (Clf) for the other staphylococci (9)], although without direct participation in platelet activation.

In sepsis, microthrombi form in the blood capillaries (10). As with IE, this phenomenon was attributed to the inflammatory environment that promoted platelet aggregation. The study by Osterud et al. also supports this hypothesis, since the authors show that in severe sepsis, the circulating monocytes show an increased expression of tissue factors, which thus support platelet aggregation (11).

Beginning in 2005, publications describing the role of platelets in immunity put prior observations related to the direct interaction of platelets and bacteria back in the spotlight (12). They then suggested that the binding of bacteria to platelets should even be considered a factor in the immune response. Today, the growing number of studies based on the inflammatory potential of platelets show that these cells express a variety of receptors, soluble molecules, and signaling factors (both hemostatic and inflammatory), enabling them to secure their position as direct effectors of antibacterial defense. This function is presented in the following sections.

#### Inflammatory and thrombotic role of platelet microparticles

Platelets also form the link between thrombosis and inflammation through the production of microparticles. Platelet microparticles (PMP) are phospholipid vesicles (100–1000 nm) that are released after budding from the platelet plasma membrane. As a result, PMP express the same antigens as their parent cells, i.e., GPIIb–IIIa, GPIb, CD31, CD61, and CD62P. This distinguishes them from microparticles derived from other cell types (red blood cells, leukocytes, monocytes, endothelial cells). PMP thus make up between 70 and 90% of the circulating vesicles. PMP differ from exosomes by their size, but also due to the fact that they are not derived from exocytosis of multivesicular bodies (13).

Platelet microparticles are released by the activated platelets in apoptosis or senescence. A central factor in the induction of this event is the detachment of the actin cytoskeleton from the plasma membrane, which occurs primarily through the increase in intracellular calcium concentration. The calcium then interacts directly with the proteins involved in proteolysis of the cytoskeleton, such as calpain (14, 15). The formation of PMP may also occur independent of calcium, in which case it involves the C5B–9 complement factor and activation of protein kinases, such as calmodulin (16).

Bacterial infection also appears to be a source of PMP formation. This has already been well described during the involvement of platelet Toll-like receptor (TLR) 4 (17–19) but also in response to the Shiga toxin (20).

Platelet microparticle formation results in an asymmetrical distribution of the membrane phospholipids. The circulating PMP thus express phosphatidylserines (PS), which are highly pro-coagulant phospholipids, on their surface (21). PMP also express tissue factor, the major initiator of the coagulation cascade (22–24). PMP have also been described as a surface that enables the *in vitro* generation of plasmin; this ability was not found in microparticles isolated from endothelial cells (25).

Platelet microparticle are also capable of issuing immunomodulatory factors, such as regulated on activation, normal T cell expressed and secreted (RANTES) (26), interleukin (IL)-1β (18), and CD40Ligand (CD40L) (27), and can also modulate the activation of inflammatory cells such as neutrophils (28, 29). PMP even seem to be able to exert their pro-inflammatory activity outside of the blood compartment (30). The pro-inflammatory function of PMP is referred to in greater detail throughout this manuscript.

Finally, the proportion of PMP in the circulation is increased in some illnesses, such as cardiovascular disease (22), sepsis (31), or HIV infection (32), which suggests they may be involved in the pathophysiology of these diseases.

### Mechanisms of interaction between platelets and bacteria

Three mechanisms of interactions between bacteria and platelets have been described to date: (1) the indirect binding of bacteria to a plasma protein, which itself is a ligand of a platelet receptor; (2) the direct binding of bacteria to platelet receptors; and (3) the binding of secreted bacterial products, particularly toxins, to platelets. The mechanisms of interaction are made more complex by the diversity of platelet receptors involved in bacterial recognition.

#### Role of glycoprotein IIb–IIIa

In addition to ensuring their usual function in hemostasis, the platelet glycoproteins play a role in the adhesion to bacteria. The first platelet receptor identified as such was GPIIb–IIIa. This integrin, specifically from the megakaryocyte cell line, is the receptor for fibrinogen. Its involvement results in adhesion and platelet aggregation (33, 34).

**Figure 1:**
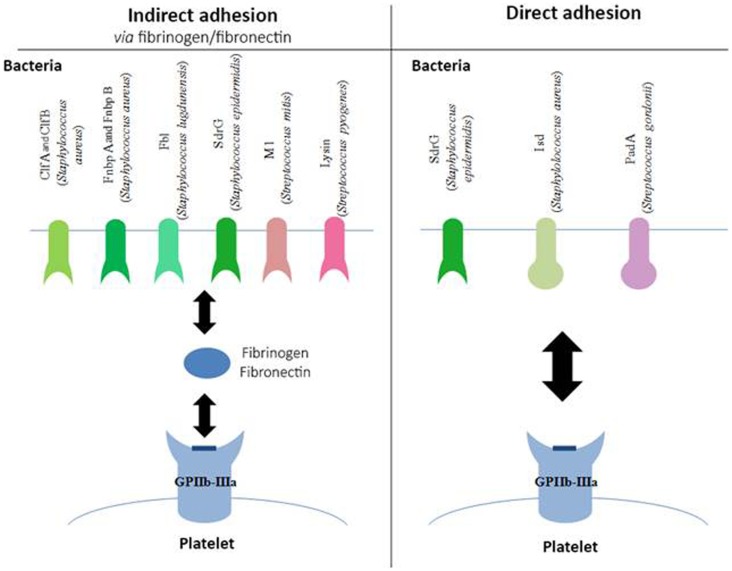
**Involvement of GPIIb–IIIa in the adhesion of bacteria to platelets**. Schematic representation of different bacterial components, which bind to the platelet GPIIb–IIIa either indirectly via fibrinogen or fibronectin (left side of the diagram) or directly (right side of the diagram). Clf, clumping factor; Fnbp, fibronectin-binding protein; SdrG, serine–aspartate repeat protein; Isd, iron-regulated surface determinant; Pad, platelet adherence protein.

*Staphylococci* express surface receptors that are specific for fibrinogen and fibronectin (Figure 1). These are surface proteins characterized by regions rich with serine–aspartate repeats, belonging to the microbial surface components recognizing adhesive matrix molecules (MSCRAMM) family (35). These molecules enable bacteria to adhere to tissues, a critical step in the establishment of infection. Some of the most common examples of MSCRAMM are found in *S. aureus*: ClfA (36), ClfB (37), fibronectin-binding protein (Fnbp) A, and FnbpB (38). *Staphylococcus lugdunensis* binds to fibrinogen via its Fbl protein, which is 58% identical to ClfA (39).

The binding of *Staphylococcus epidermidis* was long unknown, even though it had already been shown that trypsin treatment in a culture of *S. epidermidis* prevented its adhesion to platelets (40), suggesting the involvement of a membrane factor. In 2009, Brennan et al. showed that serine–aspartate dipeptide repeat (Sdr) G proteins expressed on the surface of *S. epidermidis* are necessary for the adhesion of bacteria to platelets via fibrinogen (41).

Even though these various MSCRAM are highly similar proteins, they bind to fibrinogen via different binding sites. ClfA and Fbl, as well as FnbpA and B, bind to the C-terminal region of the fibrinogen γ-chain. ClfB has its binding site on the C-terminal region of the fibrinogen α-chain, and SdrG on the β-chain. Other types of bacteria can also bind to fibrinogen, particularly *Streptococcus pyogenes (S. pyogenes)* via the M1 protein, and *Streptococcus mitis* via the enzyme, lysine (42).

More recently, bacteria have been described that also express surface proteins, enabling them to bind directly to GPIIb–IIIa, independent of fibrinogen (Figure 1). Such is the case for SdrG from *S. epidermidis*, which in addition to binding fibrinogen, can also directly target the platelet glycoprotein (41).

*In vivo*, *S. aureus* must find a source of iron that will enable it to grow and ensure its pathogenicity. To do so, it expresses iron-regulated surface determinant (Isd) proteins that are capable of binding the heme from hemoglobin and internalizing it. Yet, it has been shown that IsdB in particular can bind to GPIIb–IIIa in the absence of plasma protein. This adhesion is inhibited in the presence of platelets that have been pre-incubated with anti-GPIIb–IIIa antibodies, and in bacterial strains mutated for IsdB, confirming the specificity of the binding (43). *S. gordonii* also expresses a platelet adherence factor that has been recently described, platelet adherence protein A (PadA), and for which no other known function has been found to date (44).

The binding site(s) involved with GPIIb–IIIa have still not been mapped; however the use of peptides mimicking the arginine–glycine–aspartic acid chain, the ligand usually described for the involvement of the glycoprotein in hemostatic conditions, prevents the direct attachment of bacteria on platelets (42). This observation suggests that the bond may be the same type as with fibrinogen.

#### Role of glycoprotein Ibα

GPIbα is a membrane glycoprotein and is also only found in the megakaryocyte cell line. It belongs to the family of leucine-rich repeat proteins. It is capable of binding several ligands and is essential in primary hemostasis through its high affinity with von Willebrand factor (vWF). It is important to remember that GPIbα is found as a complex with GPIb β, GPIX, and GPV at a ratio of 2:2:2:1 (34).

**Figure 2:**
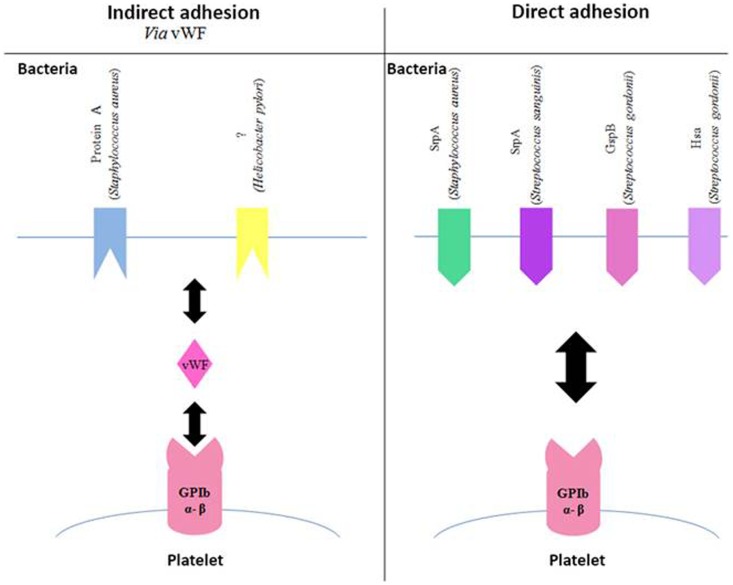
**Involvement of GPIb in the adhesion of bacteria to platelets**. Schematic representation of different bacterial components, which bind to the platelet GPIb either indirectly via the von Willebrand Factor (vWF, left side of the diagram) or directly (right side of the diagram). Gsp, glycosylated streptococcal protein; Srp, serine-rich protein; Hsa, sialic acid-binding hemagglutinin.

It has been shown that several species of *Streptococcus* are able to bind directly to GPIbα (Figure 2). This interaction involves a family of highly glycosylated, serine-rich bacterial proteins. This family includes serine-rich protein A (SrpA) from *S. sanguinis* (45), as well as glycosylated streptococcal protein B (GspB) and hemagglutinin salivary antigen (Hsa) from *S. gordonii* (46). These bacterial proteins, which are highly similar, bind to the sialic acids of the host’s receptors. The staphylococcal accessory regulator (Sar) P protein expressed by *S. aureus* also allows adhesion to platelets (47). The fact that SrpA and GspB are molecularly very close has led to the hypothesis that the SrpA–platelet bond could involve GPIbα.

Bacterial proteins are capable of binding to vWF, although they are fewer than those binding to fibrinogen (Figure 2). It has been shown that protein A from *S. aureus* is capable of binding to vWF (48–50), which in turn interacts with GPIb β. The same applies to a surface protein of *Helicobacter pylori*, though it has still not been completely characterized. This study on the platelet–bacteria interaction involving vWF shows that if vWF is already bound to bacteria, it does not require shear forces to adhere to GPIb β (51).

#### Complement receptors

**Figure 3:**
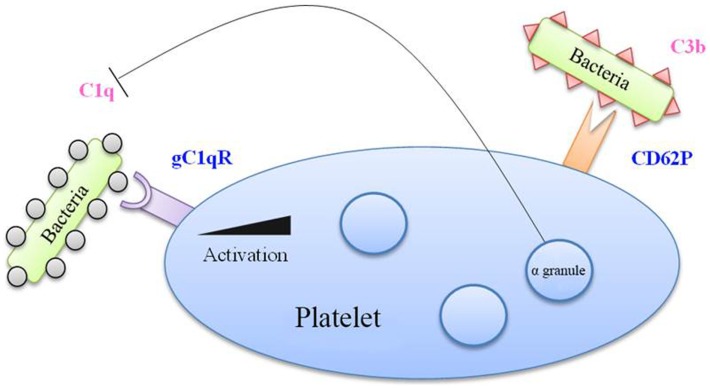
**Complement factors in bacterial binding to platelets**. Bacteria coated with C1q factor recognized by the gC1q receptor (gC1q-R) on platelet membrane, which is overexpressed upon platelet activation. Moreover, the CD62P marker, expressed by activated platelets, is able to recognize the C3b factor. Besides, alpha-granules contain a C1q inhibitor, which limits the interaction between C1q and gC1q-R.

The literature describes platelets’ ability to interact with the complement system. This is mainly observed in activated platelets, thereby allowing their clearance, but also in platelets in a pathological environment (Figure 3). For example, C5b–9 is found at detectable levels on the surface of platelets in 14% of patients with coronary artery disease. From a molecular perspective, complement is capable of activating platelets by inducing the expression of pro-coagulant factors, such as prothrombinase complex, on the surface of the cells (52).

Complement proteins also interact with bacteria both through the conventional pathway and the alternative pathway (53, 54). *S. sanguinis* for example induces platelet aggregation involving complement (55). ClfA and ClfB from *S. aureus* also induce aggregation that is dependent on complement (56).

Platelets express gC1q-R, the receptor of C1q, and could thus serve as a receptor for bacteria coated with these complement factors. Following platelet activation, the expression of gC1q-R on the platelet surface is significantly increased (57). Platelet activation also leads to the increase of CD62P at the membrane, which has been reported to bind C3b, another complement protein (58).

This interaction of platelets with complement can be bivalent. On the one hand, platelets can assist in the destruction of bacteria by increasing the activity of complement, but since they bind complement proteins, they themselves can become the target of complement’s lytic activity. This is notably what occurs in the case of thrombocytopenic purpura (52). Platelets however possess a C1 inhibitor in their α-granule, which, during platelet stimulation, would enable complement activation to be modulated (59).

This concept of platelet–bacteria binding by complement molecules involves a mechanism that is more immunologic than hemostatic, which therefore highlights the dual function of platelets.

#### FcγRIIa receptor

The expression of this immune receptor, which recognizes the Fc domain of immunoglobulin (Ig)G, is typically described for phagocytes, such as neutrophils and monocytes. FcγRIIa enables the binding and internalization of immune complexes involving IgG, whether soluble or cellular. This mechanism is regulated by the fact that complexed IgG have a strong affinity for the receptor, while it is very weak for monomeric IgG (60).

Blood platelets also express FcγRIIa (61), and it is the only type of Fcγ receptor that has been described on platelets to date. One of the first functions associated with platelet FcγRIIa involves its role in the pathophysiology of autoimmune disorders. In cases of heparin-induced thrombocytopenia, Reilly et al. described autoantibodies that recognized the platelet factor-4 (PF4)–heparin complexes binding to the platelet FcγRIIa. The involvement of the receptor then results in strong hemostatic activation, followed by clearance of the activated platelets (62).

Immunoglobulin G bound to bacteria are also capable of being taken by this platelet receptor. As with leukocytes, the immune complexes that bind FcγRIIa can even be internalized by the platelets (63).

The stimulation of other platelet receptors by bacteria very often requires the simultaneous involvement of FcγRIIa in order to obtain an effective platelet response. This suggests a link between the involvement of FcγRIIa and the mechanisms of aggregation.

On average, platelets express approximately 5,000 copies of FcγRIIa (42). Considering the large number of circulating platelets, these are thus the richest reservoir of FcγRIIa, and indeed, they are therefore a significant cell in the antibacterial platelet response.

### Bacterial toxins

Bacteria may also secrete toxins that are capable of activating platelets. *Porphyromonas gingivalis* secretes a family of cysteine proteases called gingipains. These toxins are capable of recognizing the platelet protease-activated receptor (PAR) 1 and cleave it in a manner similar to that of thrombin, thereby making it functional (64).

Alpha-toxin, expressed by strains of *S. aureus*, binds to the lipid bilayer membrane of platelets to form a pore, followed by a flow of calcium, similar to that induced by calcium ionophore (65). Other toxins capable of forming pores on the platelet surface have also been described. These include streptolysin O from *S. pyogenes* (66) and pneumolysin from *Streptococcus pneumoniae* (67).

*Staphylococcus aureus* and *S. pyogenes* produce a superfamily of toxins called staphylococcal superantigen-like (SSL) toxins, which have a known superantigenic effect. Of them, SSL5 interacts directly with GPIbα via the sialyl lactosamine residues that terminate its glycan chain. This toxin also has a direct affinity for GPIV (42).

## Effects of Bacteria on Platelet Function

Most studies focusing on the adhesion of bacteria to platelets show that it is a result of aggregation. The scope of this manuscript does not include coagulation; only the characteristics of aggregation and inflammation after bacterial contact are addressed.

### Internalization of bacteria

During systemic bacterial infection, pathogens are generally captured by phagocytes. When Clawson studied the interaction between platelets and bacteria in the 1970s, he occasionally observed the internalization of *S. aureus* in some platelets (12, 68, 69). Youssefian et al. (70) confirmed these observations, which concerned the internalization of *S. aureus* in particular. Electron microscopy photographs show the internalization of *S. aureus* in platelets, within vacuoles that are independent of the open canalicular system (OCS), suggesting active internalization.

Platelets have a better capacity for internalization when they are activated by a conventional agonist [adenosine diphosphate (ADP) or thrombin], which underscores a common mechanism between activation and internalization. Immunohistochemical labeling of vacuoles containing *S. aureus* shows the presence of CD62P and GPIIb–IIIa, but not GPIb, the phenotype that corresponds to that of an activated platelet membrane. The vacuole may therefore be formed through invagination of the plasma membrane (endocytosis) after activation.

A Japanese team confirmed the internalization of *S. aureus* in platelets but only after activation of the latter by ADP. The same study shows that *P. gingivalis* can also be internalized in platelets. There appears however to be a different mechanism of internalization at work in both bacteria. Indeed, *P. gingivalis* is capable of inducing it alone, without the addition of another platelet agonist, as the platelet aggregates are adequate for internalization of the bacteria (71).

*Staphylococcus aureus* and *P. gingivalis* share a common element with regard to internalization, as both types of bacteria are internalized in the vacuoles independent of the OCS (71). Although the final result is the same, it is thus possible that Gram-positive and Gram-negative bacteria may have different internalization mechanisms, suggesting that one of them has an additional molecule promoting its internalization. It must be emphasized that on the pictures from the study by Li et al., the presence of some *P. gingivalis* cells at the OCS may be explained by passive trapping of bacteria during platelet aggregation (71).

Platelet FcγRII may also initiate the internalization of IgG–pathogen complexes (63). One study indeed shows that platelets are capable, after involvement of FcγRII, of internalizing polystyrene beads (0.5–1.5 μm diameter) covered with IgG. This internalization is inhibited by cytochalasin D, suggesting the need to remodel platelet actin for bacteria to be internalized (72).

There remains a question as to the outcome of the internalized bacteria. White et al. reviewed arguments favoring the inability of platelets to degrade/kill bacteria. Their main argument is the absence of phagolysosomes in platelets (73). The internalization of bacteria in platelets would enable them to escape the immune system. Platelets could however use another pathway for destroying bacteria. Indeed, the endosome containing the pathogens has the ability to merge with the alpha-granules containing many bactericidal molecules (70). Finally, it is possible for *Escherichia coli* (*E. coli*) to be destroyed by platelets via internalization by FcγRII, provided that the bacteria have first been opsonized by IgG (72).

The fate of the bacteria internalized in the platelets thus remains a subject of discussion, being either a means of defending the host or an escape mechanism for the bacteria. Without progressing to internalization however, adhesion of the bacteria or bacterial products on the platelet surface is sufficient for inducing a defense response from the platelets. The main reactions are described in the following section.

### Platelet activation by bacteria

#### Effect on aggregation

Since bacterial binding to platelets includes receptors that are also involved in hemostasis, some data show aggregation to be dependent on bacteria. Bacteria that indirectly use GPIIb–IIIa (via fibrinogen or fibronectin) bring about aggregation similar to that observed with other fibrinogen-coated surfaces (42). However, when there is direct adhesion between the bacteria and GPIIb–IIIa, a different mechanism is used, and aggregation induction is often controversial in the literature. *S. gordonii*, for example, which binds directly to GPIIb–IIIa through its PadA protein, does not induce aggregation (44), potentially due to a weaker affinity for the receptor.

Bacteria that bind to GPIb via vWF can (contrary to soluble or immobilized vWF) cause aggregation in the absence of shear force. This is the case for *S. sanguinis* and *S. gordonii*, which bind to platelets through their SrpA and GspB proteins, respectively. Deletion of these two proteins completely eliminates this aggregation (42). These bacterial components are capable of substituting for the shear forces, particularly by themselves ensuring platelet rolling.

For other bacteria, *S. pyogenes* and *S. aureus* in particular, shear forces are not necessary to induce the thrombus formation, but the observed aggregation may involve other platelet proteins. The hypothesis issued by Cox et al. is that the binding of bacteria to GPIb might bring them close to functional platelet receptors such as FcγRIIa or GPIIb–IIIa (42).

After stimulation by *S. sanguinis*, which involves GPIb, the platelets release the contents of their dense granules that contain vasoactive substances, including the adenosine nucleotides, adenosine triphosphate (ATP) and ADP. Once released, ATP is taken by the ecto-ATPases present on the surface of *S. sanguinis* and hydrolyzed to ADP. The newly formed ADP, as well as that released previously, will bind to their platelet receptor. The P2Y pathway is therefore involved (42).

The use of aspirin during the adhesion of *S. sanguinis* to platelets totally inhibits aggregation, also suggesting the role of cyclooxygenase and the production of thromboxane (Tx)A_2_. The platelets exposed to *S. sanguinis* produce TxA_2_, and the TPα receptor then amplifies platelet activation by binding the newly released TxA_2_.

Platelet activation induced by *S. sanguinis* may also involve the MAP kinase pathways. McNicol et al. recently showed that MAP kinases Erk2 and p38 underwent the triphasic stages of phosphorylation/dephosphorylation observed in other phosphoproteins. Aspirin has no effect on phosphorylation and dephosphorylation of Erk2 but is able to inhibit its rephosphorylation stage (74).

There is little data available concerning platelet signaling related to the adhesion of *S. aureus*. Cox et al. showed that during the interaction of *S. aureus* with platelets, the induced aggregation is dependent on the cyclooxygenase and Tx pathways (75). This study focused mainly on the mechanisms of interaction, and therefore further details on the intracellular mechanisms were not provided.

Several studies on platelet aggregation after bacterial adhesion have also highlighted the need for FcγRIIa involvement if there is to be an effective response (42). However, FcγRIIa functioning could differ from that which is usually described, since, although the observed aggregation requires FcγRIIa, IgG does not seem to be essential (76). The colocalization of FcγRIIa with GPIbα during bacterial stimulation might be the first step in signal transduction (76, 77).

One of the most accepted hypotheses concerning the alternative role of FcγRIIa is based on platelet remodeling, since the GPIb sequence that binds to FcγRIIa (R542G543R544) is the same that binds to actin during platelet activation (77).

A similar type of study was conducted on FcγRIIa and GPIIb–IIIa. Newman et al. showed that Src residue from GPIIb–IIIa, capable of ensuring a role of tyrosine kinase, phosphorylates the immunoreceptor tyrosine-based activation motif (ITAM) residue of FcγRIIa and thus amplifies the platelet activation signals (78). Phosphorylation of the ITAM motif may take place within 30 s following contact, which shows that secondary signaling pathways can be established very quickly.

Aggregation induced by bacteria is different from that observed with the conventional ADP, ATP, and thrombin platelet agonists. It is “binary” aggregation, meaning that aggregation is not observed below a certain bacterial density, and aggregation is already at a maximum when above that density (79).

The lag time required before the appearance of aggregation is another parameter that differs according to whether the platelet stimulation is bacterial or not. This lag time is generally longer with bacterial stimulation than with hemostatic activation. Although about 10 s are needed with a hemostatic agonist, some bacteria can have a very quick lag time of 90–120 s, while others may need more than 20 min before inducing platelet aggregation. Increased bacterial density can decrease this time to a limited extent (79). Several hypotheses have been proposed to explain the lag time variations according to the bacteria: (1) the time required for platelets to bind bacteria, particularly if it done indirectly; and (2) activation of the receptor, which may not be as strong.

Bacteria-induced platelet aggregation nevertheless remains controversial, and several studies show that bacterial stimulation may not result in aggregation but a more targeted inflammatory response [chemokines release, leukocyte activation, neutrophil extracellular trap (NET) formation]. Based on current knowledge, particularly on platelet TLR, other platelet responses to bacteria, in addition to aggregation, may include the release of adapted molecules.

#### Effect on the release of immunomodulatory factors

Platelets possess many bioactive molecules in their alpha-granules, including cytokines/chemokines, which are released during their activation and enable them to act during the immune response (80, 81). The Canadian team that partners with our laboratory and studies intraplatelet signaling after *S. sanguinis* stimulation also performed a study on the secretion of platelet cytokines. Four strains of *S. sanguinis* and one of *S. gordonii* were used for the study.

The observation of aggregation and phosphorylation of signaling molecules such as PCg2 and Erk confirms the pro-thrombotic role of streptococci and also shows the release of RANTES, PF4, soluble (s)CD40L, and platelet-derived growth factor (PDGF-AB). CD62P is only released in the presence of one strain of *S. sanguinis* (82). Another study had also shown that platelets were capable of releasing soluble CD40L (sCD40L) and RANTES after stimulation with IgG-bead complexes, with the absence of aggregation and a very weak expression of CD62P (72).

Epinephrine, known to stimulate fibrinogen binding and aggregation (83), causes the opposite effect on the release of platelet cytokines. The mechanism of inhibition used is unknown but could be linked to the activation of type 3 nitric oxide synthase (NOS) after involvement of the β_2_ adrenoreceptors; the resultant generation of nitric oxide (NO) and cGMP has already been described in platelet activation inhibition (82). This clearly demonstrates that platelet aggregation and exocytosis of immunomodulating molecules are two independent functions.

One other platelet cytokine that is important in the antibacterial response is PF4. In heparin-induced thrombopenia syndrome (HIT syndrome), PF4 links to heparin through its positive charge, and together they form a neoantigen that is recognized by IgG. Likewise, soluble PF4 can bind to bacteria and thus form a new recognition site for IgG and the effector immune cells (84). This is particularly seen in Gram-negative bacteria, since PF4 presents an affinity for bisphosphorylated lipid A of lipopolysaccharide (LPS) bacteria. The newly formed complex is taken up the phagocytic cells. Platelet PF4 might thus facilitate the clearance of certain bacteria.

Finally, CD40L is the lead immunoregulatory molecule of platelets. It has been found that over 95% of plasma sCD40L originate from platelets (85). By comprising the main source of this molecule, platelets become an indisputable immunoregulatory factor; they participate both in the activation of the effector cells of innate immunity and that of adaptive immunity, since CD40L is involved in immunological synapses, as well as in class switching of B lymphocytes.

Following platelet activation, CD40L is first exposed to the membrane in trimeric form (the biologically most active form) and is then cleaved by proteolytic activity. Matrix metalloprotease (MMP)-9 is currently thought to be the most likely candidate. It should also be pointed out that the soluble form of platelet CD40L may also have an autocrine effect due to the presence of CD40 on the platelet surface (4, 86).

The involvement of CD40L in the full range of platelet functions is substantiated throughout this manuscript. This molecule is particularly implicated in the platelet response to bacteria, since the involvement of platelet TLR-2 (87–91) and TLR-4 (87, 88, 90–99) by means of their bacterial ligands, can specifically modulate the release of sCD40L. As with leukocytes, the following triad is thus observed: bacterial stimulation–platelet–detected release of immunoregulatory molecules. Hence, the role of platelet sCD40L in the pathophysiology of sepsis is an expanding field of study.

The platelet immunoregulatory molecules presented above are of great interest in the context of the immune system activation, leading to bacterial elimination. The growing number of studies on the interaction of bacteria and platelets shows that platelets can also directly influence the elimination of microorganisms through the release of bactericidal molecules.

#### Release of antibacterial platelet molecules

Kraemer et al. recently showed that platelets incubated with *S. aureus* limit the growth of this microorganism (100). The smallest platelet concentration for obtaining a bacteriostatic effect may even be possible to determine, though this would vary depending on the bacterial species. A 2013 study conducted on 17 volunteers was able to determine a critical platelet concentration for pathogens from the human oral cavity: *E. faecalis* (resistant or non-resistant to vancomycin), *C. albicans*, *S. agalactiae*, *S. oralis*, and *P. aeruginosa* (101). It should be noted that the growth of *P. aeruginosa* was not inhibited by platelet-rich plasma (PRP).

Despite these very recent studies, the first demonstration of the antibacterial role of platelets occurred very long ago. As Yeaman recounts in his literature review on the subject, Fodor already reported in 1887, the bactericidal effect of heated sera (102). The thermostable molecule involved was then identified and named β-lysine. Its platelet origin is based on the fact that it is released in coagulated plasma and is not found in the other blood cells.

Yeaman et al. were particularly interested in this platelet function and established terminology that classified these antimicrobial platelet molecules as platelet microbicidal proteins (PmP) (102), also known as thrombocidins. PmP, of which there are two (PmP1 and PmP2), are released under the induction of thrombin or bacteria, and differ from classically described defensins by their molecular mass, their sequence, and the chaining of lysine and arginine residues, which gives them a cationic charge. To become functional, these molecules must be cleaved by thrombin; the two sub-units then act in an autonomous but complementary manner by alternating the permeability of the bacterial wall (102).

The platelet signaling pathways that lead to the release of PmP depend primarily on the ATP/ADP pair and the P2 receptors. The signal is amplified through the release of ADP, and autocrine activation of the platelets is produced, which can even extend to neighboring platelets (103).

The PMP family was enlarged through the integration of kinocidins, which includes the platelet cytokines that have a direct bactericidal effect (104). They are divided into two subgroups according to the nomenclature of the cytokines. Les α-kinocidins include the CXC-cytokines [PF4, platelet basic protein (PBP), connective tissue activating peptide (CTAP3), and neutrophil activating peptide (NAP2)], while the β-kinocidins are the CC-type (RANTES). These molecules even have a synergistic effect among themselves. For example, CTAP3 does not have an effect on the viability of *E. coli*, but the presence of PF4 potentiates its activity and thereby reduces the bacterial density by 2 logs. This result is not obtained for PF4 alone (105). Structural biochemical analyses identified the 60–74 structural domain in PF4 as being responsible for the bactericidal activity (106).

Kinocidins are integrated in the mechanisms of innate immunity, to the degree that they conserve their primary role, which is the chemoattraction of leukocytes, enabling cooperation between platelet and leukocyte factors in bacterial clearance (107, 108).

In addition, Kraemer et al. showed the presence of human β-defensins 1 (hBD-1) in megakaryocytes and platelets at the level messenger RNA (mRNA) and peptides (100). Platelet hBD-1 is thus released in response to the alpha-toxin of *S. aureus*; it is not released however in response to thrombin, thrombin receptor activating peptide, or platelet-activating factor (PAF), suggesting that hBD-1 release is independent of degranulation. hBD-1 may therefore not be found in the alpha-granules, especially since the authors did not observe colocalization of the markers of these granules and of hBD-1 (100).

While Kraemer et al. were unable to observe the expression of mRNA coding for hBD-2 and -3 in the platelets, other studies have demonstrated their presence through ELISA, Western Blot, and immunohistochemistry, as well as their microbicidal activity (109, 110). As a result, it appears that platelets are involved in the infectious immune response, both directly through the release of antimicrobial factors, and indirectly through the release of cytokines, enabling them to modulate the cell-mediated immune response.

## Platelets–Bacteria: Focus on *Staphylococcus aureus* Infection

There are many studies on the interaction of platelets with *Streptococcus*, particularly oral streptococci, while comparatively few concern *S. aureus*. In 2005, a prospective study was conducted in 39 medical centers throughout 16 countries that included 1779 patients with IE. The final analysis showed that *S. aureus* was the most common pathogen implicated, with 31.6% of cases versus 18% of *Streptococcus viridans* (111). Invasive infections by methicillin-resistant *S. aureus* generally tend to spread in health care centers and result in a high level of mortality (112). It is therefore of interest to consider the role of *S. aureus* on platelets.

**Figure 4:**
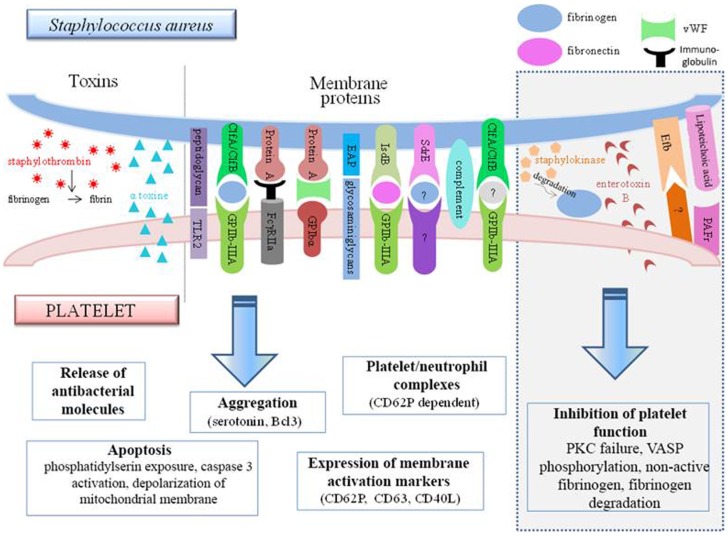
**Interconnections between *S. aureus* and platelets**. *S. aureus* can induce platelet activation by several ways, e.g., through toxin release or by using membrane protein that bind platelet receptors either directly or indirectly. However, some bacterial factors induce the inhibition of platelet function (gray frame on the right side of the diagram). TLR, Toll-like receptor; Clf, clumping factor; FcγR, Fcγ receptor; EAP, extracellular adherence protein; Isd, iron-regulated surface determinant; Sdr, serine–aspartate repeat protein; Efb, extracellular fibrinogen-binding protein; PAFR, platelet-activating factor receptor.

Most of the studies focus on molecules involved on both sides during the adhesion of *S. aureus* to platelets (Figure 4). We first looked at the interactions between the molecules present in the membrane of *S. aureus* and platelets.

Protein A, which is a surface protein of *S. aureus*, can be identified by anti-*S. aureus* antibodies. The immune complexes formed can attach to the FcγRII of the platelets, resulting in serotonin release and platelet aggregation. This reaction is dependent on the stimulation time and the quantity of immune complexes formed. The activation was found to be optimal at 5 min, and from two bacteria per platelet (75). In a more recent study, protein A was found to be incapable of inducing aggregation by itself, but it was able to maintain it (113).

Protein A from *S. aureus* can also attach to vWF, which binds to GPIbα. The use of an antibody to block vWF partially inhibits the platelet activation by *S. aureus* (79) providing evidence of the involvement of several adhesion pathways.

Clumping factor A from *S. aureus* is also involved in its attachment to platelets via fibrinogen (114). An alternative receptor exists however, since *S. aureus* adheres to platelets through the intermediary of fibrinogen/fibronectin, which does not necessarily involve GPIIb–IIIa (115). This could be shown by the persistence of aggregation, even if the two fibrinogen-binding sites on GPIIb–IIIa are blocked beforehand. It is not impossible for bacteria to transform fibrinogen so that it can be recognized by another receptor. This might thus be a method for the bacteria to increase their pathogenicity.

FcγRII and GPIIb–IIIa both have a functional role in the adhesion of *S. aureus*. The interconnection between these two receptors explains why the aggregation induced by *S. aureus* is dependent on FcγRII. This hypothesis also reflects the fact that the involvement of FcγRII alone is not sufficient for inducing aggregation, since its presence is meant to optimize the functionality of GPIIb–IIIa (79).

The identification of genetic mutations of *S. aureus* and the expression of candidate proteins in *L. lactis* (non-aggregative bacteria) showed that ClfB and the SdrE protein are also involved in platelet aggregation. The results also confirm the involvement of ClfA, which is moreover the first factor, before ClfB, that leads to aggregation, since it is the protein inducing the shortest lag time. Aggregation induced by both these factors can be inhibited by GPIIb–IIIa antagonists, aspirin, or prostaglandin E_1_ (75)_._ Aggregation is also seen with filtered platelets, suggesting a direct link between *S. aureus* and platelets, independent of fibrinogen. Different results have been observed for the SdrE protein. In order to attach to platelets, the bacterial protein requires the presence of a plasma protein other than fibrinogen, which has yet to be identified.

An important element to take into consideration is that *S. aureus* does not express the same factors depending on its stage of growth, which could be a bias in *in vitro* studies. ClfA is the dominant pro-aggregant protein in the stationary phase of growth, whereas in the exponential phase, FnBP expression dominates (79). As a result of these indications, it becomes difficult, for example, to evaluate the results of Fitzgerald, which confirm his modeling of the interaction between FnBP and platelets on bacteria in the stationary phase (116).

The complement system is important in platelet aggregation induced by *S. aureus*. It is capable of substituting for ClfA. In this instance, the lag time will be longer (between 8 and 20 min), but simultaneous involvement of the Fcγ receptor remains necessary (56).

The *Staphylococcus* protein, IsdB, could promote the adhesion and internalization of bacteria within platelets in the presence of fibronectin (117). In addition, this protein, unlike the IsdA and IsdH proteins, might induce platelet aggregation (117).

Studies on the interaction between platelets and *S. aureus* began in the 1970s and still continue today. The extracellular adherence protein, EAP, in the form of an oligomer, can bind directly to the glycosaminoglycans of platelets (118). This leads to the stimulation of thiol isomerase in the platelet and resultant platelet activation, ranging from stabilization of the fibrinogen binding, to the membrane expression of platelet activation molecules, such as CD62P, CD63, and CD40L.

In addition to having to integrate the alternative role of hemostasis receptors when confronted with *S. aureus*, there are also newly described platelet receptors that increase the range of functions. This particularly applies to TLR-2, which, by responding to the peptidoglycan of *S. aureus*, results in platelet activation after 30 min in association with a process of apoptosis characterized by depolarization of the mitochondrial membrane, exposure of PS to the plasma membrane, and caspase 3 activation.

In addition to the membrane components and the bacterial wall, *S. aureus* toxins can also modulate the platelet response (Figure 4). It has been shown *in vitro* that the alpha-toxin can interact with the platelet membrane and induce the production of microbicidal proteins and lysis of bacteria (102). This toxin might also be capable of generating dose-dependent platelet aggregation (119). It can also lead to the *de novo* synthesis in the platelet of B-cell lymphoma-3 (Bcl_3_) (119), a protein involved in the withdrawal of the platelet plug. This exotoxin is also the source of the formation of many platelet–neutrophil complexes via CD62P, an activation marker expressed on the platelet surface. Formation of the complexes increases the activation of neutrophils, which can be measured through the increase of CD11b. These aggregates could thus participate in the destruction of alveolar capillaries and be the cause of *S. aureus* hemorrhagic pneumonia (120).

*Staphylococcus aureus* releases staphylocoagulase and vWF-binding protein. Both of these molecules bind to prothrombin and form the enzymatic complex known as staphylothrombin (121). Staphylothrombin has no direct action on platelet activation, but by transforming fibrinogen to fibrin, it plays a role in stabilization of the aggregation, as well as in the initiation of secondary activation.

It has been noted, however, that several *S. aureus* molecules seem to actually inhibit the hemostatic function of platelets. In this respect, staphylococcal enterotoxin B (SEB) has been observed to cause platelet overactivation of protein kinase C (PKC). This enzyme, essential for platelet response, is therefore no longer found in physiological conditions and therefore cannot ensure its function. This explains why platelets incubated with SEB are incapable of ensuring correct aggregation in response to thrombin (122).

Lipoteichoic acid uses the PAF receptor to increase the level of cAMP within platelets. This latter then increases its phosphorylation activity on vasodilator-stimulated phosphoprotein (VASP) and inhibits aggregation and thrombus formation (123). The anti-thrombotic role of *S. aureus* can also be attributed to the extracellular fibrinogen-binding protein (Efb). *In vitro*, this protein has been described as adhering to platelets (on a non-characterized receptor or on fibrinogen). Once attached, it recruits fibrinogen but in a non-conventional form that is rather inclined to inhibit platelet activation. The inhibitory action of this molecule was confirmed *in vivo*, in which it is able to prevent thrombosis following treatment with platelet agonists (124).

Finally, staphylokinase also exerts inhibitory activity on the platelets although indirectly. This enzyme degrades plasmin and fibrinogen, thereby preventing aggregation (125). Several groups are also studying the interaction of *S. aureus* and platelets under conditions similar to those seen *in vivo*, particularly with regard to preservation of the necessary shear forces. Mice infected by *S. aureus* develop thrombi through a ClfA-dependent mechanism (126). The use of a molecule occupying the binding site of ClfA on fibrinogen completely prevents aggregation, which demonstrates the predominance of ClfA, despite the multitude of other factors present in the microenvironment.

Dogs with *S. aureus* infection quickly develop sepsis, accompanied by platelet dysfunction (reduction in the capacity of growth in response to a PAR-4 agonist). This latter result suggests that beyond their role in IE due to *S. aureus*, platelets participate in the pathophysiology of *S. aureus*-induced sepsis (127).

In addition, a duality can be seen in the effect of *S. aureus* bacteria on platelets, indicating the complexity of the interaction. All of the studies are based on whether the bacteria have pro-aggregant or non-aggregant abilities. There is no data however on the release of platelet cytokines, which is nevertheless a very important component of bacterial infection.

## Role of Platelets in the Pathophysiology of Sepsis

### Sepsis and coagulopathy

Data from the early 2000s show that between 30 and 50% of patients with severe sepsis have disseminated intravascular coagulation (DIC), resulting in organ hypoxia (128). During the inflammatory response, the neutrophils release tissue factors that trigger the coagulation cascade, leading to platelet activation. IL-1 and IL-6 are also strong inductors of coagulation (129, 130). This phenomenon is amplified by deregulation of the anticoagulant balance. Patients with sepsis have a strong release of PAI-1, a natural plasmin inhibitor. There is also a reduction in protein C, the active form of which is an inhibitor of coagulation factors Va and VIIIa (10, 129–133). In addition, these natural anticoagulants have their role in thrombin generation, with anti-inflammatory properties influencing nuclear factor κB (NFκB) (134).

Reactive oxygen species (ROS) released in massive quantities during the acute phase of sepsis are also responsible for coagulopathies. In mice with induced sepsis that are knocked-out for NOS (the enzyme that produces ROS), vasoconstriction is reduced compared to wild mice. By favoring vasoconstriction, ROS participates in circulatory alteration in the blood capillaries. ROS also have a direct effect on the hemostatic activation of platelets (132).

The fourth element favoring excessive coagulation is the increase of adhesion factors. In endotoxemia models, the expression of adhesion molecules is increased in both the platelet and endothelial membranes. The adhesion of platelets to the endothelium promotes mutual activation and an accumulation of platelets, resulting in vessel occlusion (118, 123). Furthermore, in endotoxemia induced in a mouse model, it was seen that overexpression of the endothelial PAI-1 molecule in the lungs limits the *in situ* recruitment of regulatory T lymphocytes but promotes that of neutrophils (135).

Sepsis-related coagulation disorders have highlighted the role of platelets in the pathophysiology of sepsis, particularly through their hemostatic function. However, it is now clear that platelets also possess an inflammatory function that may enable them to directly participate in the amplification of inflammation associated with the early phases of sepsis. In addition to their sensitivity to thrombin, adhesion molecules, and cytokines/chemokines, the hypothesis of their direct involvement in sepsis is also supported by the TLR expression on their surface and the broad range of inflammatory molecules that they can release during bacterial stimulation.

### Sepsis and inflammatory platelet molecules

The first elements supporting the participation of platelets in sepsis-associated inflammation come from studies showing that the level of circulating sCD40L is greater in patients with sepsis than in individual controls (age- and gender-matched) but independent of the severity of the sepsis (136–141). This sCD40L released during sepsis comes from the platelets, since mice that have undergone platelet depletion do not present this increased plasma level (142). One recent study suggests that matrix metalloproteinase-9, which is also increased during sepsis, might be the source of platelet CD40L cleavage (143). This molecule has important inflammatory properties affecting many cells, both immune and non-immune, and may significantly amplify inflammation (144, 145).

In sepsis, sCD40L participates in the recruitment of neutrophils. CD40L gene-deficient C57BL/6 mice that had sepsis induced through cecal ligation puncture do not show neutrophil activation, edema formation, or neutrophil infiltration in the lungs, and they maintain their alveolar microarchitecture (142, 146).

Soluble CD40L may enable the expression of the macrophage-1 antigen (Mac-1) adhesion protein by neutrophils, promoting their recruitment at the mucosa. The mechanism of action of sCD40L may involve macrophage inflammatory protein 2 (MIP-2) and its receptor, CXCR2. This is supported by the fact that *in vitro* recombinant sCD40L does not increase the expression of Mac-1 on the neutrophils in culture (142).

Platelet-activating factor is a molecule synthesized by various cells and that possesses a cytokine function. Its receptor, platelet-activating factor receptor (PAFR), is attached to G proteins and is expressed by the cells that participate in immune defense and coagulation, including platelets. Platelet PAFR activation results in the release of inflammatory factors, degranulation, and the initiation of coagulation cascades. In physiological conditions, signaling associated with PAFR is finely regulated in order to avoid an excessive thrombo-inflammatory response. During sepsis however, this regulatory balance is disrupted, and PAF is then involved in the activation of neutrophils, monocytes, platelets, and in the formation of leukocyte–endothelium, leukocyte–platelet, and platelet–endothelium complexes (147).

A recent study suggested an association between the duration of storage of apheresis platelets before transfusion and the occurrence of complications in patients from a trauma center. This study was conducted on 381 patients who had been admitted to a trauma center and received apheresis platelet concentrates that had been stored for 3 days or less, 4 days, or 5 days. The results show that the transfusion of platelets stored for over 3 days may increase the risk of complications for the patient, sepsis in particular (148). It was shown that during the storage of platelet concentrates, the platelets are activated, and the platelet inflammatory factors, including sCD40L, are released and then accumulate (149–152). This suggests that the inflammatory molecules that accumulate during the storage of platelet concentrates could promote the onset of sepsis.

It has also recently been shown that IL-27 could be a predictive molecule of sepsis in children (153), and that the activated platelets could be a significant source of this cytokine (154). Thrombin formed during sepsis could lead to this release of platelet IL-27.

In addition, an increase in the level of circulating microparticles has been reported in septic patients. These vesicles (granulocytes, monocytes, endothelial cells, and platelets) may arise from several cell types (22). In sepsis, the release of PMP is accompanied by an increase of CD62P at the platelet membrane and an increase of platelet–monocyte aggregates (31). The role of microparticles in sepsis requires further exploration. It has been described however that they possess strong pro-coagulant pathogenicity through the expression of tissue factors. Some studies conducted in other contexts have shown that PMP are rich in CD40L (155) and IL-1 β (18, 30), which are two pro-inflammatory molecules that are strongly associated with the pathophysiology of sepsis. It therefore becomes important to consider PMP in the development of inflammation during sepsis.

The role of platelets in the inflammatory phase of sepsis may not be limited to the production of inflammatory molecules. Indeed, in patients with uncomplicated sepsis, the level of circulating platelet–leukocyte complexes is higher than in controls. However, in sepsis complicated by organ failure, the number of platelet–leukocyte complexes is decreased. This can be explained by sequestration of the complexes in the damaged organs, for example the lungs (156). The same type of observation occurred *in vitro*, in which strains of *S. aureus* isolated from bacteremia result in aggregation, the formation of platelet–neutrophil complexes, and activation of these neutrophils (157).

Moreover, since platelets are capable of binding to bacteria, and even keeping them alive intracellularly, they could promote their dissemination within the body. This mechanism was proposed in a mouse model infected by *S. pyogenes* via the intraperitoneal route. Platelet-depleted animals are unable to ensure the transport of bacteria in the blood, lungs, and spleen, which is characterized by a reduction in the bacterial load (through a CFU count) in these organs after sacrifice of the animals (158).

### Platelet apoptosis in the microenvironment of sepsis

The involvement of platelets in sepsis is also characterized by persistent thrombocytopenia in patients (159, 160). There have been various studies done that consider thrombocytopenia a predictive factor of the mortality rate of patients admitted to intensive care (160, 161). Several hypotheses have been made with regard to the decrease in circulating platelets and are presented below.

During sepsis, platelets express activation factors that promote their sequestration in the spleen and then their destruction (162). Platelet depletion can be accelerated if the platelet–bacteria contact involves the complement system (52) or Fc-γ receptor (163). Furthermore, sepsis is a pathological state that might promote hemophagocytosis of platelets by macrophages which is partly dependent on macrophage colony-stimulating factor (164).

The failure of thrombopoiesis is an unlikely hypothesis to the degree that the plasma levels of IL-6, tumor necrosis factor (TNF)-α, and thrombopoietin are increased in sepsis (129, 130, 165). On the contrary, the involvement of megakaryocyte TLRs might rather promote an overproduction of platelets.

Another hypothesis points at sepsis-induced coagulopathy leading to DIC, in which disseminated thrombi may immobilize the platelets (166). The results of Tyml et al. confirmed this in a mouse model of sepsis (132).

More recently, the scientific community has been interested in platelet apoptosis. This mechanism of cellular death involves an important step of nuclear transformation involving chromatin condensation, followed by DNA fragmentation (167–169).

Apoptosis, which is known as “programed cell death,” can normally be triggered by two types of stimuli. These are referred to as the intrinsic pathway and the extrinsic pathway.

The extrinsic pathway involves cell death receptors, such as the apoptosis stimulating fragment (Fas) receptor, the ligands of which are the proteins from the TNF family. The involvement of Fas causes trimerization of the receptor, which then becomes active, enabling it to recruit an adaptor molecule, Fas-associated protein with death domain (FADD), which also contains a binding domain for procaspase-8. The formation of this complex leads to cleaving of caspase-8, which is then produced in its active dimeric form. Caspase-8 will then either activate the sequential cascade of the different caspases, or the previously described mitochondrial pathway.

The intrinsic pathway is triggered following cellular stress, such as oxidative stress. Under physiological conditions, Bcl_2_ protein and other similar proteins maintain the integrity of the mitochondrial membrane. Under stress conditions, these proteins are degraded. Transition pores then form on the mitochondrial surface, causing them to swell and then burst. Cytochrome C is then released in the cytoplasm and can activate the caspase cascade (170).

In both cases, the pathways converge to activate caspase-3, which has many substrates. The effects of caspase-3 are seen both on the nuclear proteins involved in DNA repair and on cytoplasm proteins such as gelsolin, which is a cytoskeletal regulator. Cleaving of the molecules involved in cellular structure and repair causes DNA and cytoskeletal fragmentation, but the plasma membrane remains intact. During the formation of apoptotic bodies, a change in the distribution of phospholipids is produced, termed “membrane flip flop,” which exposes the PS on the membrane surface. The PS constitute an “eat me” signal for the phagocytic cells, which enables the elimination of the apoptotic bodies (170).

Platelets have also been found to possess apoptotic machinery. The platelets express caspases 1, 3, 4, and 9. Caspases 2, 6, and 8A have a more reduced expression, and no expression for caspases 5, 7, and 10 has been demonstrated. Fas are not expressed in the platelets, but they have other cell death receptors, such as DR3, DR5, TNF-receptor p55, and RIP. Platelets also express proteins from the Bcl-2 family: Bcl-X, Bfl1, Bad, Bak, Bax, and Mc1 (171).

Activation of these apoptotic molecules has been demonstrated during storage of platelet concentrates. After 5 days of storage in standard blood bank conditions, platelet viability decreases. This platelet death is accompanied by an increase in PS at the cell surface and an increase in caspase-3 activity. Caspase activation occurs in a specific manner, since the use of an inhibitor (z-VAD-fmt) stops this process. Platelet death is thus well associated with a specific apoptosis (171).

This first study showed the independence of apoptosis and platelet activation, since PS exposure was independent of the increase in the expression of the CD63 activation marker, and thrombin activation had no effect on caspase-3 activity. The scientific community however is not in unanimous agreement. It has even been shown that thrombin stimulation induced depolarization of the mitochondrial membrane, the expression of pro-apoptotic molecules (Bax, Bak), activation of caspase-3, and exposure of PS to the membrane (172). The action of thrombin may not be direct and may require the release of platelet factors that can induce apoptosis. During storage, platelets release many soluble factors, including TNF-α, which is a strong inductor of apoptosis through the induction of caspases.

The life span of platelets could therefore be controlled through apoptosis. Caspase-9, for example, is required for platelet and megakaryocyte death but is not involved in their production or their functionality (159).

The Bcl-X family is also at the center of platelet survival. These molecules may even determine their life span. Two missense mutations of Bcl-X are capable of accelerating platelet death resulting in severe thrombocytopenia (173).

It has also been shown that a strain of isolated *E. coli* in a patient with sepsis was able to induce apoptotic manifestations of platelets, such as actin condensation, a decrease of the mitochondrial potential, and degradation of Bcl-X. The use of mutant and non-pathogenic strains showed that only pathogenic strains that release toxins forming pores have the ability to degrade Bcl-X. This applies to the alpha-toxin of *E. coli* and *S. aureus*. These toxins probably act on calpain, since this protein is the source of Bcl-X degradation. However, proteasome inhibition is ineffective in preventing degradation of the pro-apoptotic protein (174). This study is the first demonstration that pathogenic bacteria can influence the intrinsic initiation of platelet apoptosis. The degradation of Bcl-X suggests a new mechanism by which bacteria may be able to cause the thrombocytopenia observed in patients with bacteremia. This mechanism could then explain why depolarization of the mitochondrial membrane potential (ΔΨm) is increased in the platelets of patients with SIRS (175). A decrease in ΔΨm could even be associated with SRIS progression.

### Involvement of platelets in sepsis-related NETose

Another phenomenon, the formation of NETs involving platelets, neutrophils, and bacteria, has been demonstrated and could play an important role in sepsis. It was first described in 2004. Neutrophils that have been activated, particularly by IL-8, release their granular (peptides and enzymes) and nuclear (chromatin and histones) components in the microvessels where they combine to form a network called NET (176). The use of high-resolution electron microscopy has confirmed the structure of NET, which is characterized by extracellular chromatin stretches that are associated with globular proteins. It has also been shown that a bacterial environment, or that mimicked through the injection of LPS, results in the *in vivo* release of NET that are able to trap bacteria and thus reduce their spread during sepsis (177).

It was noted that bacterial trapping at NET is greater under flow conditions, as in the blood circulation (94). NET can reach a diameter of 25 nm, and once combined, they form a structure that can be over 100 nm both in diameter as well as length (94).

Aligned with DNA, NET contain the histones H1, H2A, H2B, and H4, as well as granular proteins such as elastase, myeloperoxidase, and bactericidal permeability increasing protein, which enable bacterial degradation (176). NET are able to stop both Gram-positive and Gram-negative bacteria. The histones and BPI have a proteolytic action on the alpha-toxin of *S. aureus*, as well as on the IpaB of *S. flexneri* (177). *C. albicans*, although not a bacteria, is also sensitive to NET, but its destruction may only depend on granular proteins and not histones (177).

Neutrophil extracellular trap release occurs within 5–10 min after stimulation of the neutrophils. This time period is too short for the implementation of an apoptosis or necrosis mechanism. It is therefore an active mechanism and not the consequence of disintegration of the neutrophilic plasma membrane (176).

From a molecular viewpoint, the formation of NET involves (178, 179):

- peptidylarginine deiminase type 4, for chromatin decondensation;
- ROS formation, which is NADPH oxidase-dependent for disintegration of the nuclear membrane;
- actin cytoskeleton and microtubules for NET release.

Neutrophil extracellular trap can be released according to three mechanisms (180): (1) a rapid mechanism (30–60 min) involving vesicles; in this case, the neutrophils remain viable; (2) a slower mechanism (3–4 h), resulting in rupture of the neutrophilic plasma membrane; or (3) directly from the mitochondria. At present, the third mechanism and the existence of NET composed from mitochondrial DNA remains controversial, although one study shows that NET might be majorly composed of mitochondrial DNA rather than nuclear DNA (181).

Lipopolysaccharide, which has traditionally been described as a neutrophil activator, is surprisingly incapable of inducing *in vitro* NET release by neutrophils. In contrast, in 2007, Clark et al. showed in a mouse model that the intravenous injection of LPS leads to NET formation within the first 5 min (94). A more detailed investigation showed that LPS-induced NET formation is not direct and requires platelet participation. Indeed, platelet stimulation by LPS in the presence of neutrophils may not cause a standard platelet response but might promote their adhesion; the neutrophils would then be activated and form NET (94).

Kraemer et al. then showed that type 1 beta-defensins released by platelets after bacterial stimulation were responsible for NET formation (100).

Kubes presents platelets as a barometer that can detect a substantial level of bacteria. The platelets are activated by an LPS concentration 100 times greater than that inducing neutrophil activation. The platelets may therefore come to the assistance of the neutrophils by enabling them to form NET when the bacterial load is too high and their normal functions are insufficient for correctly eliminating the bacteria (177). NET could be the innate “last chance” defense.

Neutrophil extracellular traps have been shown to have a positive impact on the destruction of pathogens during bacteremia. Conversely, they may alter the microvascular circulation by promoting the formation of microthrombi, thereby also preventing the immune cells from reaching the bacteria. In addition, their components may have a toxic effect on the host cells. This was confirmed *in vitro* on human umbilical vein endothelial cell (HUVEC) (94). Hepatotoxicity has been observed *in vivo* following the release of NET, which was measured by the release of alanine aminotransferase and an occlusion of the liver sinusoids (94).

The histones released in the extracellular medium possess pro-thrombotic activity and are capable of activating the platelets via their TLR-2 and -4 (90). This phenomenon may be extrapolated to the NET histones. In this case, NET would be responsible for platelet overactivation, which may lead to thrombi formation. The potential detrimental effect of NET is another illustration of the alteration of platelet function during sepsis.

## Conclusion

The implication of platelets in the inflammatory response marks a veritable turning point in the understanding of platelet physiology and opens new fields of investigation that have been up to now somewhat neglected. The identification of platelets in the various inflammatory mechanisms places them at the center of innate immunity, whether in the recognition of pathogens, signal transduction, or the release of cytokines/chemokines. This functional similarity with leukocytes shows that both of these cells types are not so different.

Anucleated platelets are only found in mammals. In the lower vertebrates, such as birds, reptiles, amphibians, or fish, hemostatic function is ensured by nucleated thrombocytes. With regard to invertebrate species, they do not possess platelets *per se*, but the hemolymph contains a type of nucleated cell called a hemocyte that expresses TLR, which is capable of phagocytizing the foreign body or secreting antimicrobial proteins. It is this same cell however that regulates coagulation and healing. By compiling all of these characteristics, it is possible to discuss a possible common evolution between platelets and leukocytes, followed by a dissociation, proportional to the evolution of the species (1, 3, 4, 182), despite this is debated.

All of the studies presented in this thesis generally show that platelets are able to cover the majority of the steps of inflammation and thus confirm their total involvement in the orchestration of this pathophysiological state. By modulating both the acute effector phase of inflammation and the maintenance of this process, platelets may become a therapeutic target.

## Author Contributions

All authors contributed substantially to the conception of this review. HC, PD, and AC drafted it while BP, FC, and OG revised it critically for important intellectual content. All authors approved the final version and agree to be accountable for all aspects of the work in ensuring that questions related to the accuracy or integrity of any part of the work are appropriately investigated and resolved.

## Conflict of Interest Statement

The authors declare that the research was conducted in the absence of any commercial or financial relationships that could be construed as a potential conflict of interest.

## Abbreviations

Bcl, B-cell lymphoma; C1q, first complement component; CD40L, CD40Ligand; Clf, clumping factor; CTAP3, connective tissue activating protein-3; DIC, disseminated intravascular coagulation; *E. coli*, *Escherichia coli*; Fas, apoptosis stimulating fragment; Fnbp, fibronectin-binding protein; gC1q-R, gC1q receptor; Gsp, glycosylated streptococcal protein; hBD-1, human beta-defensin-1; IE, infectious endocarditis; Ig, immunoglobulin; IL, interleukin; Isd, iron-regulated surface determinant; ITAM, immunoreceptor tyrosine-based activation motif; LPS, lipopolysaccharide; Mac-1, macrophage-1 antigen; mRNA, messenger RNA; MSCRAMM, microbial surface components recognizing adhesive matrix molecules; NET, neutrophil extracellular trap; NO, nitric oxide; NOS, nitric oxide synthase; OCS, open canalicular system; *P. gingivalis*, *Porphyromonas gingivalis*; PAF, platelet-activating factor; PAFR, platelet-activating factor receptor; PAR, protease-activated receptor; PF4, platelet factor-4; PMP, platelet microparticles; PmP, platelet microbicidal proteins; PS, phosphatidylserines; RANTES, regulated on activation, normal T cell expressed and secreted; ROS, reactive oxygen species; *S. aureus*, *Staphylococcus aureus*; *S. epidermidis*, *Staphylococcus epidermidis*; *S. gordonii*, *Streptococcus gordonii*; *S. pyogenes*, *Streptococcus pyogenes*; *S. sanguinis*, *Streptococcus sanguinis*; sCD40L, soluble CD40L; Sdr, serine–aspartate dipeptide repeat; SEB, staphylococcal enterotoxin B; SrpA, serine-rich protein A; SSL, staphylococcal superantigen-like; TLR, Toll-like receptor; TNF, tumor necrosis factor; Tx, thromboxane; vWF, von Willebrand factor; ΔΨm, mitochondrial membrane potential.
